# Coupling CDH17 and CLDN18 markers for comprehensive membrane-targeted detection of human gastric cancer

**DOI:** 10.18632/oncotarget.11638

**Published:** 2016-08-26

**Authors:** Keisuke Matsusaka, Tetsuo Ushiku, Masayuki Urabe, Masaki Fukuyo, Hiroyuki Abe, Shumpei Ishikawa, Yasuyuki Seto, Hiroyuki Aburatani, Takao Hamakubo, Atsushi Kaneda, Masashi Fukayama

**Affiliations:** ^1^ Division of Diagnostic Pathology, The University of Tokyo Hospital, Tokyo, Japan; ^2^ Department of Molecular Oncology, Graduate School of Medicine, Chiba University, Chiba, Japan; ^3^ Department of Pathology, Graduate School of Medicine, The University of Tokyo, Tokyo, Japan; ^4^ Department of Gastrointestinal Surgery, Graduate School of Medicine, The University of Tokyo, Tokyo, Japan; ^5^ Genome Science Division, Research Center for Advanced Science and Technology, The University of Tokyo, Tokyo, Japan; ^6^ Department of Quantitative Biology and Medicine, Research Center for Advanced Science and Technology, The University of Tokyo, Tokyo, Japan

**Keywords:** gastric cancer, intratumoral heterogeneity, nodal metastases, CDH17, CLDN18

## Abstract

Patients with gastric cancer typically face gastrectomies even when few or no nodal metastases are reported. Current procedures poorly predict lymphatic metastases; thus, evaluation of target molecules expressed on cancer cell membranes is necessary for *in vivo* detection. However, marker development is limited by the intratumoral heterogeneity of gastric cancer cells. In this study, multiple gene expression arrays of 42 systemic normal tissue samples and 56 gastric cancer samples were used to investigate two adhesion molecules, *cadherin 17* (*CDH17*) and *claudin 18* (*CLDN18*), which are intestinal and gastric markers, respectively. Expression of *CDH17* and *CLDN18* was partially redundant, but overlapped in 50 of 56 cases (89.3%). Tissue microarrays constructed using primary lesions and nodal metastases of 106 advanced gastric cancers revealed CDH17 and CLDN18 expression in 98 positive cases of 106 (92%). Hierarchical clustering classified gastric cancers into three subgroups, CDH17(++)/CLDN18(+/−), CDH17(++)/CLDN18(++) or CDH17(+)/CLDN18(+), and CDH17(−)/CLDN18(++/+/−). Whole tissue sections displayed strong, homogeneous staining for CDH17 and CLDN18. Together, these results indicate that CDH17 and CLDN18 are useful target molecules; moreover, their coupling can aid in the comprehensive detection and localization of gastric cancer metastases *in vivo* to overcome challenges associated with intratumoral heterogeneity.

## INTRODUCTION

Gastric cancer is the third leading cause of cancer-related death worldwide [1]. Although gastrectomy with lymphadenectomy is a first-line choice for more complicated cases [2], nodal dissection often extends to non-metastatic lymph nodes [3]. These overtreatments are attributed to the absence of effective methods to detect cancer cells *in vivo* before or during an operation. The classical concept of the sentinel lymph node (SLN) is defined as the first node encountered by the lymphatic flow from the primary lesion and detected by intraoperative injection of a dye or radioactive tracer [2]. However, the complex distribution of lymphatic flow through the stomach makes detection of SLNs quite difficult [2]. Therefore, target molecules developed to detect metastases *in vivo*, such as in the intraoperative imaging method [4–6], could lead to limited operations and result in relief of complications after gastrectomy. Recent technological advances have enabled the visualization of target molecules *in vivo* with intraoperative fluorescence imaging procedures [7–12]. However, comprehensive and specific membrane-targeting molecules for gastric cancer have not been recognized [13–15] because gastric cancer often shows intratumoral heterogeneity as well as variance among individual cases [16]. In practice, trastuzumab combined with chemotherapy was approved for HER2-overexpressing gastric cancers, but the HER2 expression pattern is often not homogeneous, thwarting effective therapy [15, 17, 18].

To detect lymph node metastases by intravenous injection of a specific antibody binding to certain targets, candidate molecules should meet the following criteria: (1) expression on the cell membrane; (2) enriched expression in gastric cancers; (3) intratumorally homogeneous expression pattern; and (4) absence of expression in other vital organs, lymph nodes, and subserous tissue. In this study, we data-mined a published database of gene expression arrays and focused on *cadherin 17* (*CDH17*), also known as liver-intestine cadherin [19], and *claudin 18* (*CLDN18*), a component of tight junctions, for further evaluation. Subsequently, tissue microarrays (TMAs) were constructed by mounting primary lesions and nodal metastases of advanced gastric cancers. The immunostaining distribution ratio was evaluated for CLDN18 and CLDN7, which are known adhesion molecules in the stomach and intestine, respectively [20]. Subsequently, clinicopathological information was analyzed among subgroups classified by expression and immunostaining patterns. Finally, whole tissue sections of primary lesions and nodal metastases were analyzed, and homogeneous immunostainability was confirmed. These results indicate that co-detection of CDH17 and CLDN18 enables prediction of the general feature of individual cancer cases by preoperative biopsy examination and allows for comprehensive detection of gastric cancer metastases.

## RESULTS

### Data mining microarray expression data

**Figure 1:**
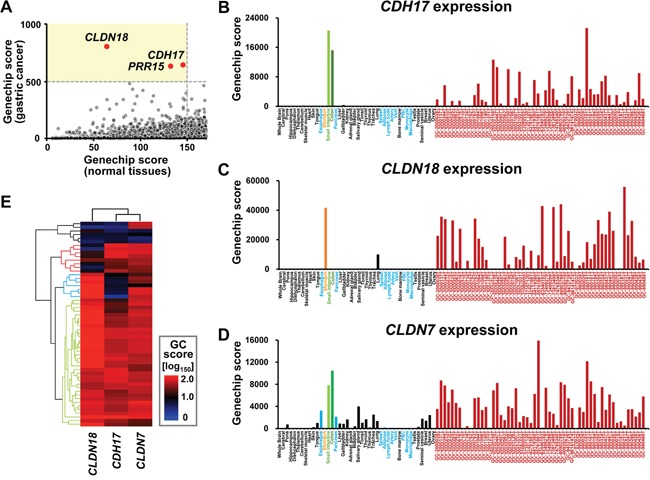
Gene expression pattern in systemic organs and human gastric cancer **A.** Scatter plot of Genechip score. The horizontal axis indicates the maximum value of representative normal tissue (esophagus, pancreas, spleen, adipose, lymph node, artery, vein, peripheral blood cells, monocyte, and macrophage without stomach, colon, and small intestine). The vertical axis represents the 40th value from the top among 56 gastric cancer cases. When the threshold for normal tissues was set to <150 fold and that for gastric cancer was ≥500 fold, represented by the light yellow area, CLDN18, CDH17, and PRG15 are selected out of 9, 131 probes. **B-D.** Systemic expression array data for *CDH17*, *CLDN18*, and *CLDN7*. Data are indicated by the following colors: normal tissue around stomach, light blue; stomach, orange; colon, light green; small intestine, green; gastric cancer, dark red; others, black. *CDH17* and *CLDN18* were frequently and specifically expressed in gastric cancer, except for colon and small intestine for *CDH17* (B) and stomach and lung for *CLDN18* (C). *CLDN7* was frequently expressed in gastric cancer. However, the gene showed less specificity because it was expressed in various normal tissues such as pancreas and esophagus (D). **E.** Unsupervised two-way hierarchical clustering by Genechip score (GC score). Patterns of expression among *CDH17*, *CLDN18*, and *CLDN7* were analyzed by hierarchical cluster analysis. The horizontal axis indicates antibodies and vertical axis indicates cases. High and low scores are shown by red and blue, respectively. *CDH17* and *CLDN7* clustered as intestinal-type features. Although expression of *CDH17* and *CLDN18* overlapped and thus was redundant (light green dendrogram), expression was also partially mutually exclusive and specific (light blue and red dendrogram). Thus, the coupling of *CDH17* and *CLDN18* as molecular markers covered gastric cancers in 50 of 56 cases (89.3%).

Candidate genes for detection of gastric cancer nodal metastases were mined from microarray expression data based on frequent and enriched expression in gastric cancer, as well as low expression in tissues around the stomach that could prevent detection of nodal metastases. Three candidate genes, *cadherin 17* (*CDH17*), *claudin 18* (*CLDN18*), and *proline rich 15* (*PRR15*), were extracted (Figure 1A). *CDH17* and *CLDN18* showed high specificity for gastric cancer cells (Figure 1B-1C). *CDH17* encodes a liver-intestine cadherin [19] expressed in the colon and small intestine (Figure 1B). *CLDN18* encodes a gastric type adhesion molecule [20, 21] expressed in the stomach and lung (Figure 1C). *CLDN7* encodes an intestinal-type adhesion molecule and was investigated as a comparative control for the other two markers. However, *CLDN7* is expressed in various normal tissues with less specificity, and could be inadequate for gastric cancer detection (Figure 1D). To further explore the relative expression patterns of *CDH17*, *CLDN18*, and *CLDN7*, hierarchical cluster analysis was performed on these genes in 56 gastric cancer cases (Figure 1E). While *CDH17* and *CLDN18* overlapped with respect to tissue localization, they also showed exclusivity. Combination of *CDH17* and *CLDN18* covered 50 cases out of 56 (89.3%), which indicated that the coupling of markers for *CDH17* and *CLDN18* provides an opportunity to detect gastric cancer using specific antibodies.

**Figure 2:**
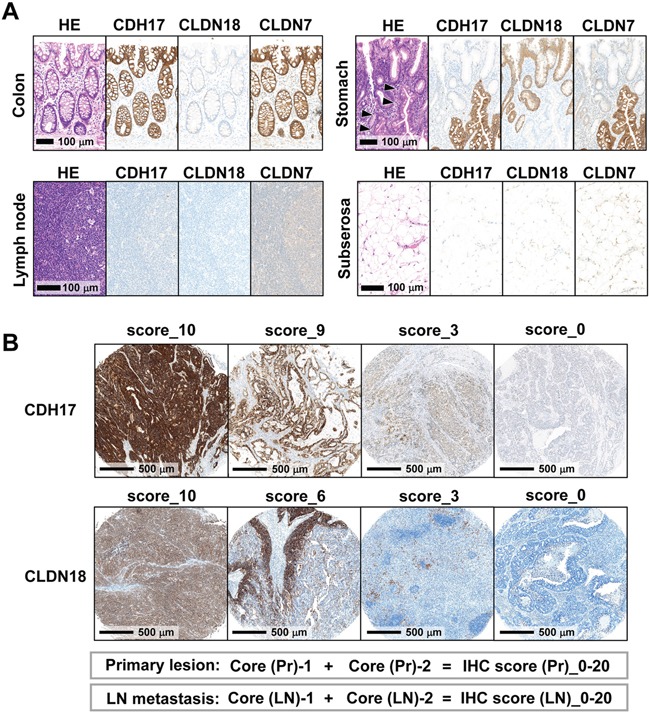
Immunohistochemical analysis of normal tissue and TMAs of gastric cancer tissue **A.** Immunostaining patterns of CDH17, CLDN18, and CLDN7 in colon, stomach, lymph node, and subserosal adipose tissue. Intestinal-type CDH17 and CLDN7 were expressed on the cell membrane of colonic and intestinal metaplastic epithelia (arrowhead). Gastric-type CLDN18 was expressed on the cell membrane of gastric foveolar epithelium. **B.** Evaluation of immunohistochemistry (IHC) scores in TMAs. Representative immunostaining patterns of CDH17 and CLDN18 with TMA cores are shown. The membranous immunostaining distribution of CDH17, CLDN18, and CLDN7 was scored as an IHC score according to the proportion of membranous positive staining among total cancerous cells on a zero-to-ten scale. IHC scores were evaluated in duplicate for both primary lesions (Core (Pr)-1 and -2) and lymph node metastases (Core (LN)-1 and -2), respectively, and summed to 20 scores (IHC score (Pr)/(LN)).

To compare expression of CDH17, CLDN18, and CLDN7 in colon, stomach, lymph nodes, and adipose tissue, immunostaining was performed (Figure 2A). Intestinal-type CDH17 and CLDN7 were expressed in colonic and atrophic gastric epithelia with intestinal metaplasia (Figure 2A). Gastric-type CLDN18 was expressed in gastric foveolar epithelium. CDH17 and CLDN18 did not display immunoreactivity in interstitial cells such as fibroblasts, immune cells, lymphatic tissue, and adipose tissue (Figure 2A).

### Clustering analysis according to immunohistochemistry (IHC) scores

**Figure 3:**
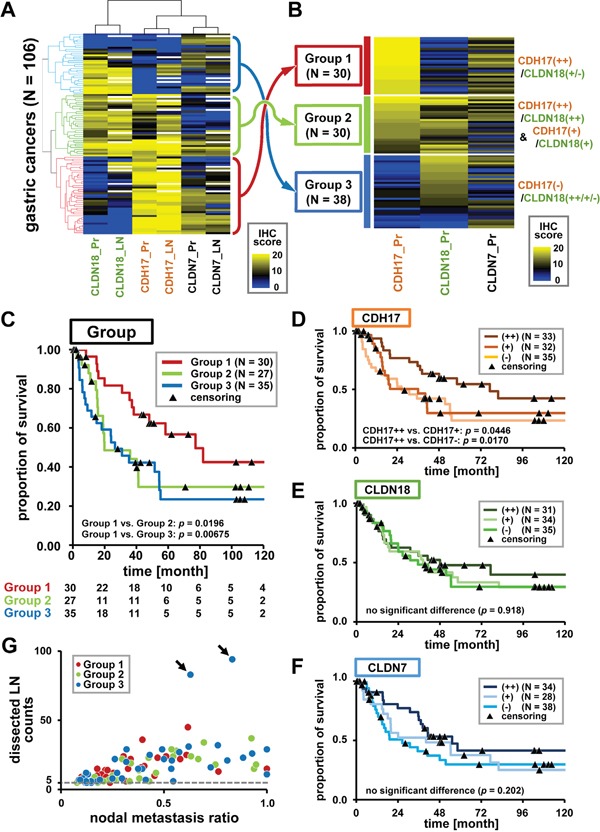
Unsupervised two-way hierarchical clustering and prognostic analyses **A.** Unsupervised two-way hierarchical clustering by IHC score. Horizontal axis indicates antibodies for duplicated primary lesion (Pr) and lymph node (LN) TMA, and vertical axis indicates cases (*n* = 106). Yellow and blue represent high and low IHC scores, respectively. Some nodal cores did not contain a cancer component and were shown by a white column. Hierarchical cluster analysis revealed a strong correlation between primary lesions and nodal metastases. A combination of CDH17 and CLDN18 provided broad diagnostic coverage (98 of 106 cases (92.5%)) with partially redundant but specific immunostaining patterns. CDH17 and CLDN18 staining enabled detection of the three major subgroups. **B.** Subgrouping by CDH17 and CLDN18 immunostaining patterns. Immunostaining patterns were defined as follows: CDH17(++)/CLDN18(++), homogenously and strongly positive; CDH17(+)/CLDN18(+), partially and moderately positive; CDH17(−)/CLDN18(−), weakly positive or negative. Gastric cancer was classified into three subgroups: Group 1 (CDH17(++)/CLDN18(+/−), red dendrogram in Figure 3A), Group 2 (CDH17(++)/CLDN18(++) and CDH17(+)/CLDN18(+), green dendrogram), and Group 3 (CDH17(−)/CLDN18(++/+/−), blue dendrogram). Groups 1, 2, and 3 contained 30, 30, and 38 cases, respectively. **C.** Prognostic analyses in Groups 1, 2, and 3. Prognostic analyses were performed in Group 1 (dark red), Group 2 (light green), and Group 3 (dark blue). Numbers at risk are shown below the graph. Group 1 (CDH17(++)/CLDN18(+/−)) demonstrated significantly better prognosis than that of Group 2 and Group 3 (*p* <0.05). **D-F.** Prognostic analysis by evaluation of CDH17, CLDN18, and CLDN7. Prognostic analyses in all cases with prognostic information (100 cases) were performed according to CDH17, CLDN18, and CLDN7 immunostainability. Cases with strongly positive immunostainability for CDH17 (CDH17(++)) showed significantly better prognosis than cases with moderate or negative CDH17 expression (CDH17(+) or (−)). (D). However, there was no correlation between CLDN18 and CLDN7 expression and prognosis (EF). **G.** Scatter plot of nodal metastasis. The horizontal axis represents the ratio of nodal metastases to total dissected lymph nodes, and the vertical axis represents the number of dissected lymph nodes. Each case in Group 1, 2, and 3 is represented by dark red, light green, and dark blue dots, respectively. Group 3 contained two extreme cases (arrows) with a high frequency of nodal metastases.

IHC scores of CDH17, CLDN18, and CLDN7 expression levels were evaluated semi-quantitatively in TMA-mounted primary lesions (IHC score (Pr)) and lymph node metastases (IHC score (LN)) taken from 106 cases of advanced gastric cancers (Figure 2B). Two-way hierarchical cluster analyses according to IHC scores were performed to determine immunostaining pattern correlations (Figure 3A). For each antibody, a strong correlation in immunostaining patterns was found between primary lesions and nodal metastases. Expression of intestinal-type CDH17 and gastric-type CLDN18 was found to be partially redundant and partially specific in representation, similar to the oligonucleotide microarray data (Figure 1E). Combination of CDH17 and CLDN18 markers detected 92.5% of gastric cancer cases (98 of 106 cases). CLDN7 showed a similar immunostaining pattern to that of CDH17, another intestinal-type marker, but the intensity was lower than that of CDH17 and CLDN18. The immunostainability of CDH17 and CLDN18 enabled detection of three major subgroups, depicted in (Figure 3A).

According to hierarchical clustering applied to the threshold of CDH17 and CLDN18 IHC scores of primary lesions, gastric cancers could be classified into three subgroups (Figure 3B). The three subgroups were defined as follows: Group 1 (dark red), CDH17 showed strong and homogeneous staining (CDH17(++) (IHC score 18–20)), and CLDN18 showed absent or weak staining (CLDN18(+/−) (IHC score 0–13)); Group 2 (light green), CDH17(++) (IHC score 18–20) and CLDN18 showed strong and homogeneous staining (CLDN18(++) (IHC score 14–20)), and CDH17 showed moderate staining (CDH17(+) (IHC score 9–17)) and CLDN18 showed moderate staining (CLDN18(+) (IHC score 6–13)); Group 3 (dark blue), CDH17 showed absent or weak staining (CDH17(−) (IHC score 0–8)) and CLDN18 showed any immunostainability (CLDN18(++/+/−)). Groups 1, 2, and 3 contained 30, 30, and 38 cases (out of 98), and 8 cases were removed without application of the criteria described above.

### Prognosis analyses

Prognosis analyses were performed on data for Groups 1, 2, and 3 with prognostic information, representing 92 cases (30, 27, and 35 cases, respectively) using the Kaplan-Meier method (Figure 3C). Group 1 showed significantly better prognosis than that of Groups 2 or 3, using a generalized Wilcoxon test (*p* <0.05). Subsequently, prognostic analyses for all cases with prognostic information (100 cases) were performed according to CDH17, CLDN18, and CLDN7 staining (Figure 3D–3F). Immunostaining patterns of CDH17 and CLDN18 were categorized as CDH17(++)/(+)/(−) and CLDN18(++)/(+)/(−) using the same criteria as in Figure 3B. CLDN7 was categorized as follows: CLDN7(++) (IHC score_12–20), CLDN7(+) (IHC score_8–11), and CLDN7(+) (IHC score_0–7). CDH17-strongly positive cases (CDH17(++)) showed significantly better prognosis than that of CDH17-moderately positive (CDH17(+)) and CDH17-weakly positive/negative cases (CDH17(−)) (Figure 3D). However, no correlation was found between CLDN expression and prognosis (Figure 3E-3F).

Clinicopathological features were investigated for each group (Table 1), and Group 1 showed a significantly lower frequency of Stage IV cases than Group 2 (*p* = 0.04). The number of nodal metastases in Group 3 appeared to be higher than that in Groups 1 and 2. However, Group 3 contained two cases with frequent nodal metastases (Figure 3G). No significant difference was revealed by the non-parametric Mann–Whitney *U* test (*p* >0.05). Univariate and multivariate survival analysis using a Cox proportional hazards model was performed for each parameter (Table 2). CDH17 hyperexpression, low stage, less number of lymph node metastases, and intestinal or mixed type histology were independent markers for better prognosis (*p* <0.05). Prognostic analyses for each parameter using the Kaplan-Meier method were also performed (Supplementary Figure S1A-S1I). Stage, T-classification, and number of lymph node metastases were found to be significant factors for worse prognosis as progression using a generalized Wilcoxon test (Supplementary Figure S1D-S1E, S1H).

**Table 1:** Clinicopathological features of gastric cancer groups

	All cases	Group 1	Group 2	Group 3	*p* value G1 vs. G2	*p* value G1 vs. G3	*p* value G2 vs. G3
**# of cases**	98 (100%)	30 (31%)	30 (31%)	38 (39%)			
**Age**							
**mean ± SD (year)**	64 ± 12.0	64.0 ± 11.5	63.0 ± 12.3	66.0 ± 12.4	0.567	0.904	0.640
**≤60 yo**	35 (36%)	10 (33%)	12 (40%)	13 (34%)	0.896	0.538	0.854
**61–70 yo**	34 (35%)	13 (43%)	10 (33%)	11 (29%)			
**≥71 yo**	29 (30%)	7 (23%)	8 (27%)	14 (37%)			
**Sex**							
**Male**	61 (62%)	18 (60%)	21 (70%)	22 (58%)	0.588	0.861	0.439
**Female**	37 (38%)	12 (40%)	9 (30%)	16 (42%)			
**Tumor size**							
**mean ± SD (mm)**	83.8 ± 40.5	77.7 ± 32.8	83.4 ± 44.4	88.9 ± 43.7	0.572	0.233	0.756
**≤50 mm**	24 (24%)	5 (17%)	9 (30%)	10 (26%)	0.569	0.389	0.915
**51-100 mm**	49 (50%)	19 (63%)	14 (47%)	16 (42%)			
**≥101 mm**	25 (26%)	6 (20%)	7 (23%)	12 (32%)			
**Tumor location in the stomach**							
**Upper**	29 (30%)	13 (43%)	8 (27%)	8 (21%)	0.282	0.227	0.742
**Middle**	36 (37%)	7 (23%)	14 (47%)	15 (39%)			
**Lower**	33 (34%)	10 (33%)	8 (27%)	15 (39%)			
**Stage**							
**IIA-B**	6 (6%)	1 (3%)	3 (10%)	2 (5%)	*0.042	0.360	0.580
**IIIA-C**	60 (61%)	24 (80%)	13 (43%)	23 (61%)			
**IV**	32 (33%)	5 (17%)	14 (47%)	13 (34%)			
**T-classification**							
**T1b, T2**	10 (10%)	3 (10%)	5 (17%)	2 (5%)	0.895	0.939	0.537
**T3**	24 (24%)	8 (27%)	6 (20%)	10 (26%)			
**T4a, T4b**	64 (65%)	19 (63%)	19 (63%)	26 (68%)			
**Lymphatic invasion**							
**ly0-1**	32 (33%)	7 (23%)	8 (27%)	17 (45%)	1.00	0.115	0.200
**ly2-3**	66 (67%)	23 (77%)	22 (73%)	21 (55%)			
**Venous invasion**							
**v0-1**	39 (40%)	9 (30%)	12 (40%)	18 (47%)	0.588	0.229	0.543
**v2-3**	59 (60%)	21 (70%)	18 (60%)	20 (53%)			
**# of LN metastases**							
**Metastatic**	1642 (36%)	465 (31%)	445 (36%)	732 (39%)	0.515	0.858	0.484
**Total**	4625	1496	1238	1891			
**Average # of dissected LN (/case)**							
	47.2	49.9	41.3	49.8			
**Histology**							
**Intestinal type**	2 (2%)	1 (3%)	0 (0%)	1 (3%)	0.921	0.842	0.908
**Diffuse type**	47 (48%)	15 (50%)	13 (43%)	19 (50%)			
**Mixed type**	49 (50%)	14 (47%)	17 (57%)	18 (47%)			

Group 1 showed a significantly lower frequency of Stage IV than Group 2. The degree of lymphatic/venous invasion was evaluated using a 4-grade scale [16]; ly0/v0, no invasion; ly1/v1, minimal invasion; ly2/v2, moderate invasion; ly3/v3, marked invasion. G1, Group 1; G2, Group 2; G3, Group 3; yo, year-old; SD, standard deviation; T1b, submucosa; T2, muscularis propria; T3, subserosa; T4a, penetrates the serosa and is exposed to the peritoneal cavity; T4b, invades adjacent structures; LN, lymph node. Student's *t*-test; age, tumor size. chi-squared test; sex, tumor location in the stomach, stage, T-classification, lymphatic/venous invasion, histology. Mann–Whitney *U* test; # of LN metastases.

* *p* <0.05.

**Table 2:** Univariate and multivariate survival analysis using Cox proportional hazard model for overall survival

Variables	Univariate	Multivariate		
	*p* value	HR	95% CI	*p* value
**CDH17**				
**[−] vs. [+] vs. [++]**	0.030*	0.640	0.425-0.963	0.032*
**CLDN18**				
**[−] vs. [+] vs. [++]**	0.549	0.810	0.541-1.214	0.308
**CLDN7**				
**[−] vs. [+] vs. [++]**	0.131	0.870	0.594-1.276	0.476
**Age**				
**[≤60] vs. [61-70] vs. [≥71]**	0.473	1.015	0.989-1.042	0.252
**Sex**				
**[Male] vs. [Female]**	0.903	1.268	0.692-2.325	0.443
**Tumor size (mm)**				
**[≤50] vs. [51-100] vs. [≥101]**	0.091	0.720	0.445-1.164	0.18
**Stage**				
**[IIA-B] vs. [IIIA-C] vs. [IV]**	<0.001**	2.493	1.284-4.837	0.007*
**T classification**				
**[T1b+T2] vs. [T3] vs. [T4a+T4b]**	0.0013**	1.787	0.966-3.309	0.064
**Lymphatic invasion**				
**[ly0-1] vs. [ly2-3]**	0.133	1.381	0.67-2.845	0.381
**Venous invasion**				
**[v0-1] vs. [v2-3]**	0.717	0.568	0.309-1.046	0.069
**# of LN metastases**				
**[5-10] vs. [11-20] vs. [≥21]**	<0.001**	1.753	1.143-2.688	0.010*
**Histology**				
**[I+M] vs. [D]**	0.211	0.506	0.272-0.941	0.032*

Multivariate survival analysis demonstrated that weak or negative expression of CDH17, advanced stage, more lymph node metastases, and diffuse-type histology are independent factors for significantly worse prognosis. HR, Hazard ratio; CI, confidence interval. I, intestinal type; M, mixed type; D, diffuse type.

* *p* <0.05,

** *p* <0.01.

### Whole-section analysis of IHC staining patterns

**Figure 4:**
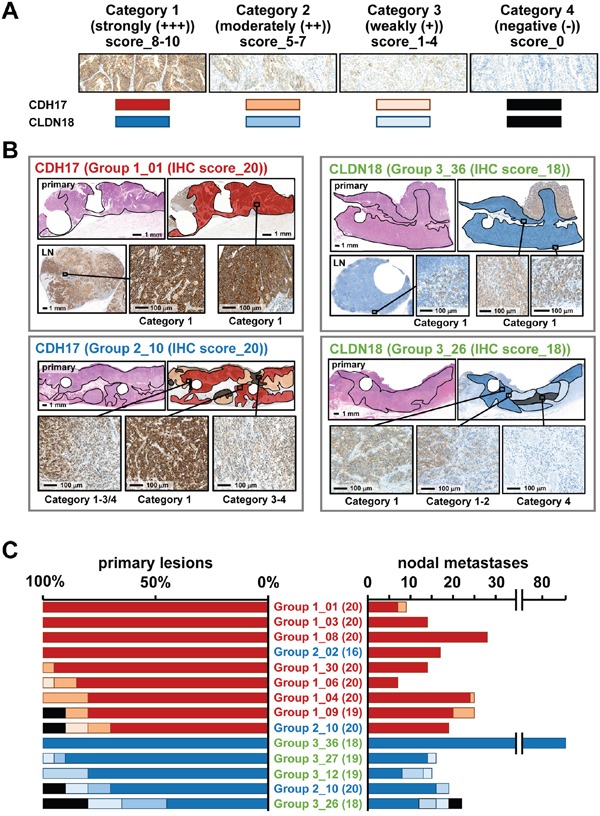
Whole section analysis of immunostaining of CDH17 and CLDN18 **A.** The proportion of staining was classified into four stepwise categories according to IHC scores: Category 1 (strongly positive (+++)) corresponding to a score of 8–10 by dark red and dark blue; Category 2 (moderately positive (++)), score of 5–7 by light orange and light blue; Category 3 (weakly positive (+)), IHC score_1–4 by pale orange and pale blue; and Category 4 (negative; absent staining (−)), IHC score_0 by both black. **B.** Schematic depiction of immunostaining quantification for CDH17 and CLDN18. Two cases each are shown for CDH17 and CLDN18. Circular defect parts of each section are where TMA cores were obtained. Left upper panel shows Group 1_01, with an IHC score (Pr) for CDH17 of 20. Whole sections in the primary lesion and nodal metastasis show diffuse and strong immunostaining of CDH17 in Category 1. The left lower panel shows Group 2_10, with an IHC score (Pr) for CDH17 of 20. Whole sections of primary lesions show heterogeneous immunostaining of CDH17 in Categories 1 to 4. The right upper panel shows Group 3_36, with an IHC score (Pr) in CLDN18 of 18. Whole sections in primary lesion and nodal metastasis show diffuse and strong immunostaining of CLDN18 in Category 1. The right lower panel shows Group 3_26, with an IHC score (Pr) in CLDN18 of 20. Whole sections of primary lesions show heterogeneous immunostaining in Categories 1 to 4. **C.** Representative cases with high IHC scores (Pr) in CDH17 and CLDN18 were evaluated in whole tissue sections. Sample names are expressed as Group-x_case number (IHC score in TMA). CDH17 showed homogeneous and strong immunostaining in most cases. Some cases showed Category 4 (negative) in CDH17; however, all nodal metastases show strong positive staining (Category 1 or 2). Although CLDN18 also showed clear immunostaining, the pattern was slightly more heterogeneous than that of CDH17 and some nodal metastases in Category 4 (negative or absent staining).

Immunostaining patterns of CDH17 and CLDN18 were investigated in whole sections of cancer tissue from representative cases (9 cases for CDH17 and 5 cases for CLDN18), including primary tumors and nodal metastases to remove some of the focal biases of TMAs (Figure 4). The proportion of immunostaining reactivity was classified into four stepwise categories according to score: Category 1 (strongly positive (+++)), score 8–10; Category 2 (moderately positive (++)), score 5–7; Category 3 (weakly positive (+)), score 1–4; and Category 4 (negative or absent (−)), score 0 (Figure 4A). For CDH17, 7 cases in Group 1 and two cases in Group 2 showed high IHC scores in TMA (≥16). Seven of 9 cases showed diffuse and moderate staining. The other two cases (Group 1_09 and Group 2_10) also showed diffuse staining despite a portion with absent staining, and all nodal metastases showed strong or moderate staining (Figure 4B-4C). For CLDN18, one case in Group 2 and four cases in Group 3 showed high IHC scores (≥18). CLDN18 appeared to show relatively diffuse staining but slightly more heterogeneity compared to CDH17. Moreover, several nodal metastases showed an absence of staining in Group 3_26.

## DISCUSSION

In this study, we selected *CDH17* and *CLDN18* through mining of systemic microarray gene expression data, based on their high frequency of expression in gastric cancer tissues and absence of expression in other major vital organs. These markers are candidates for practical membranous target molecules to detect metastases of gastric cancer *in vivo* to complement intraoperative imaging procedures. Three superior characteristics were identified using this pair of markers. Firstly, CDH17 and CLDN18 demonstrated homogeneous immunostaining in gastric cancer tissue. Secondly, just two markers, CDH17 and CLDN18, was sufficient to account for more than 90% of gastric cancer cases. Lastly, the CDH17-hyperexpression subgroup displayed significantly better prognosis than that of the other subgroups.

The most innovative point of this study is that the combination of CDH17 and CLDN18 demonstrated homogeneous and robust expression in more than 90% cases of gastric cancer, even in diffuse-type cases. Homogeneity is important not only for actual *in vivo* detection by intraoperative imaging procedures, but also for prediction of immunostaining properties by preoperative biopsy examination. According to mucin phenotypes, gastric cancer has been classified as gastric, intestinal, mixed, and null-type varieties [22]. Intestinal-type CDH17 and gastric-type CLDN18 could be a useful combination to enable coverage of most gastric cancers that show a partial redundancy but primarily specific behavior. Because previous studies focused on the heterogeneity of gastric cancer, notably concerning HER2 status [17, 18, 23–30], potentially important information regarding homogenous aspects has been neglected. The cocktail of fluorescently labeled anti-CDH17 and anti-CLDN18 antibodies in the present study could be applied to detect metastatic foci by means of intraoperative imaging procedures. In fact, we have developed an anti-CDH17 monoclonal antibody that could eventually be used in clinical settings (unpublished data). It should be noted that, CDH17-negative/CLDN18-positive cases could warrant a certain level of caution, as CLDN18 rarely showed heterogeneous staining in the primary lesion or in nodal metastases (Figure 4B-4C). Preoperative biopsy specimens should be obtained from multiple sites to avoid false negatives.

In terms of biology, CDH17 is a cell-cell adhesion molecule reported to be expressed on the cell membrane of normal small and large intestinal epithelia, atrophic gastric mucosa with intestinal metaplasia, normal pancreatic ductal epithelium, and several types of tumors, e.g., colorectal cancer, gastric cancer, pancreaticobiliary adenocarcinoma, and hepatocellular carcinoma [19, 31–33]. The characteristics of CDH17 in gastric cancer concerning prognosis or biological significance have been reported [19, 31–38], although the correlation between CDH17 expression and prognosis has been controversial [34–36]. In this study, only advanced cases with more than five nodal metastases were selected. Among the cases with the worst original prognoses, univariate analysis demonstrated that cases with CDH17 hyperexpression showed significantly better prognosis, with fewer Stage IV cases (Figure 3C-3D). Multivariate analysis confirmed that CDH17 hyperexpression property was an independent factor for better prognosis (Table 2). These results suggest that CDH17 could be utilized for a marker for better prognosis. If these procedures enable detection of metastatic foci *in vivo* during an operation in real time, then less invasive operations, such as laparoscopy and endoscopy cooperative surgery [39, 40], could be applied when no metastases are detected. In particular, cases with CDH17 hyperexpression cases have a better prognosis with less distant metastasis and represent an ideal initial application for less invasive or limited operations.

The *CLDN18* gene encodes a claudin that is a component of tight junction strands. *CLDN18* has been reported to encode a gastric type of claudin expressed specifically on the cell surface of the gastric foveolar epithelium [20]. CLDN18 is a superior marker for gastric differentiation and is broadly implicated in various tumors, including those of the ovary [21] and pancreaticobiliary neoplasms [41, 42]. Notably, CLDN18 splice variant 2 (CLDN18.2) has been reported as a pan-cancer target for therapeutic antibody [43–46], and more recently a randomized phase II trial of an anti-CLDN18.2 antibody combined with first-line chemotherapy reported a clinically relevant benefit profile in patients with CLDN18.2-positive gastric and gastroesophageal junction adenocarcinoma (American Society of Clinical Oncology annual meeting 2016, Clinical trial information: NCT01630083). Homogenous expression of CLDN18 would thus be expected to be beneficial for molecular targeted therapy. Furthermore, *CLDN18* was also found to be focused in *CLDN18*–*ARHGAP26* fusions in gastric cancer [47], which mediated epithelial disintegration [48].

In summary, we identified a useful pair of target molecules, CDH17 and CLDN18, to aid in the comprehensive detection and localization of gastric cancer metastases *in vivo* to overcome challenges associated with intratumoral heterogeneity.

## MATERIALS AND METHODS

### Ethics statement

This study was approved by the University of Tokyo Institutional Ethical Committee. Clinical samples were collected with written informed consent under the University of Tokyo Institutional guidelines for the study of human tissues.

### Data-mining for extraction of candidate genes

To extract candidate genes associated with gastric cancer, genome-wide gene expression data were analyzed using Affymetrix GeneChip Human Genome U133 plus 2.0 oligonucleotide arrays in 42 types of systemic normal tissue, as we previously reported [49]. Data were analyzed using a GeneChip Scanner 3000 (Affymetrix, Fremont, CA, USA). To obtain signal value Genechip scores for each probe set, Affymetrix GeneChip Operating Software v1.3 with the MAS5 algorithm was used. Expression array data of systemic normal tissues are available at GEO datasets (GSE43346). In addition, gene expression levels in 56 gastric cancer samples were evaluated using U133 plus 2.0 oligonucleotide arrays collected from the GEO datasets (GSE34942) [50]. In global normalization, the MAS5 algorithm was used [51]. Candidate genes were extracted based on the following criteria: Genechip score <150 fold that of normal tissues, which could prohibit detection of nodal metastases if expressed (esophagus, pancreas, spleen, adipose, lymph node, artery, vein, peripheral blood cells, monocyte, and macrophage without stomach, colon, and small intestine) and ≥500 fold in 40 gastric cancers of 56 cases (71.4%).

### Case selection for tissue microarray (TMA)

Overall, 1, 424 consecutive cases of gastric cancer involving surgical resection between 2000 and 2009 were reviewed from pathological files at the University of Tokyo Hospital. Of cases with more than five pathological nodal metastases, 295 cases were extracted. Then, hematoxylin and eosin (HE)-stained slides from these cases were reviewed by a pathologist (KM), and 106 cases containing abundant lesions in both the primary lesion and lymph node were selected for construction of TMAs. Each case was represented by duplicated 2.0 mm tissue cores of both primary lesions and nodal metastases obtained from formalin-fixed paraffin-embedded (FFPE) tissue blocks.

### Immunohistochemical studies in TMA

FFPE blocks of TMA were cut into 4-μm-thick sections and deparaffinized by xylene using an antigen retrieval procedure. Immunohistochemical analyses were performed with antibodies against CDH17 (clone #141713, 1:100), CLDN18 (polyclonal, 1:1000), and CLDN7 (clone 5D10F3, 1:200) using a Ventana Benchmark XT autostainer system [20]. A CDH17 antibody was purchased from R&D Systems (Minneapolis, MN, USA), and CLDN antibodies were purchased from Zymed (San Francisco, CA, USA). Immunostaining of CDH17, CLDN18, and CLDN7 was scored semi-quantitatively according to the proportion of positive membrane staining among total cancerous cells on a zero-to-ten scale as follows: score 0, 0%; score 1, 1%-10%; score 2, 11%-20%, score 3, 21%-30%, score 4, 31%-40%, score 5, 41%-50%; score 6, 51%-60%; score 7, 61%-70%; score 8, 71%-80%; score 9, 81%-90%; and score 10, 91%-100%. Scores were calculated as duplicate cores of both the primary lesion (Core (Pr)-1 and -2) and lymph node metastases (Core (LN)-1 and -2), respectively, presented as an immunohistochemistry (IHC) score ((Pr)/(LN)).

### Evaluation of immunostaining patterns in whole sections

Representative cases of gastric cancer were evaluated for staining patterns for each marker in whole sections of primary lesions and all lymph nodes. The evaluated markers included CDH17 (7 cases from Group 1 and 2 cases from Group 2) and CLDN18 (one case from Group 2 and 4 cases from Group 3). The tumor area of the primary lesion was categorized into four levels according to the zero-to-ten scale, as for the TMA evaluation: Category 1, strongly and homogeneously positive (+++) corresponding to a score of 8–10; Category 2, moderately and non-homogeneously positive (++) corresponding to a score of 5–7; Category 3, weakly and heterogeneously positive (+) corresponding to a score 1–4; and Category 4, negative (−) corresponding to a score of 0. The total cancer area in the primary lesion was divided arbitrarily into 10 sections and assigned to the appropriate category. Lymph node metastases were classified into representative categories for individual nodes.

### Hierarchical clustering analysis

Unsupervised two-way hierarchical clustering analysis was performed using oligonucleotide array data for gastric cancers and IHC scores of primary lesions and nodal metastases based on the City Block distance and Correlation distance with an average linkage clustering algorithm for the sample and antibody, respectively, using Cluster 3.0 software (http://bonsai.hgc.jp/~mdehoon/software/cluster/). A heat map was drawn using Java TreeView software (http://jtreeview.sourceforge.net/). For the oligonucleotide array data, Genechip scores were represented log base 150.

### Statistical analyses

Fisher's exact tests or chi-squared tests were applied to compare categorical variables among groups. Student's *t*-tests were used to compare age and tumor size. The non-parametric Mann–Whitney *U* test was used to compare the frequencies of nodal metastases. Differences were considered to be significant when *p* <0.05.

### Prognostic analyses

Prognostic analyses were performed among Groups 1, 2, and 3, and among all cases with observed immunostaining for CDH17, CLDN18, and CLDN7. Cases without prognostic data were excluded. Survival was defined as the time elapsed between the day of surgery and the day of death by primary gastric cancer. Follow-up of surviving patients was censored on the last day. Survival was analyzed using the Kaplan-Meier method, which invokes generalized Wilcoxon tests for comparison. Statistical prognostic analyses were performed using MS Excel Statistics Software 2015 (Social Survey Research Information Co., Ltd., Tokyo, Japan), and differences were considered statistically significant when *p* <0.05.

## SUPPLEMENTARY MATERIALS FIGURE


