# Supplementary material for: Coupling CDH17 and CLDN18 markers for comprehensive membrane-targeted detection of human gastric cancer
Source: Oncotarget. 2016 Aug 26;7(39):64168–81. doi: 10.18632/oncotarget.11638 (PMC5325433; doi:10.18632/oncotarget.11638)
Supplement: Supplementary file 1 [file oncotarget-07-64168-s001.pdf]

Coupling CDH17 and CLDN18 markers for comprehensive membrane-targeted detection of human gastric cancer

SUPPLEMENTARY FIGURE

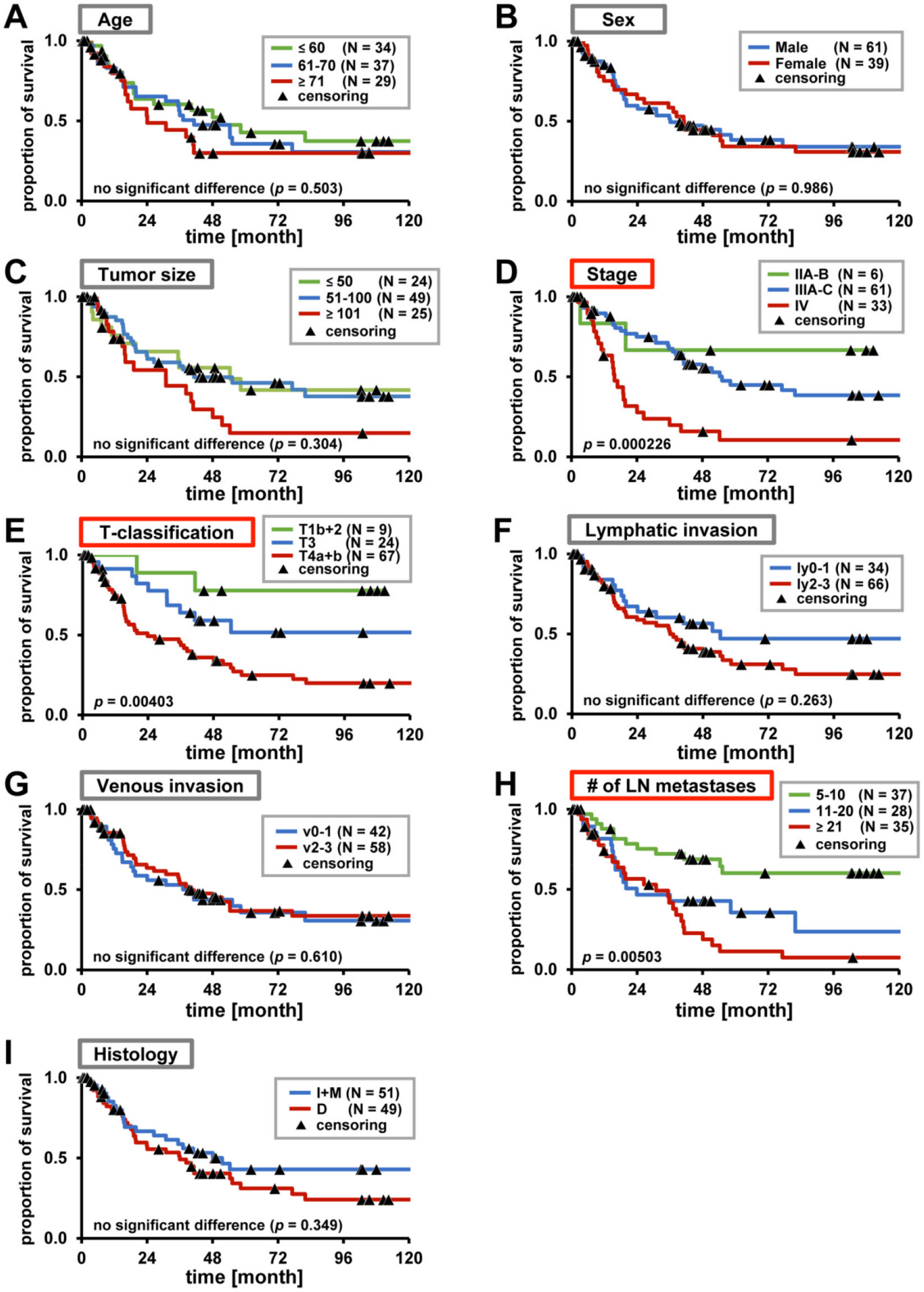

Supplementary Figure S1: Prognostic analyses for each parameter. Prognostic analyses using the Kaplan-Meier method were performed for age **A.** sex **B.** tumor size **C.** stage **D.** T-classification **E.** lymphatic invasion **F.** venous invasion **G.** and number of lymphatic metastases **H.** histology **I.** Stage, T-classification, and number of lymph node metastases were significantly associated with worse prognosis according to progression.
